# The role of residential urban form and built environment in supporting social interaction, health, and well-being: a focus on forming and maintaining ties

**DOI:** 10.1186/s12942-026-00451-z

**Published:** 2026-01-27

**Authors:** Taiyo Ishikawa, Marketta Kyttä, Tiina Rinne

**Affiliations:** 1 Department of Built Environment, School of Engineering, Aalto University, P.O. Box 14100, Aalto, 00076 Finland; 2 Transport Research Center Verne, Faculty of Built Environment, Tampere University, P.O. Box 600, Tampere, 33014 Finland

**Keywords:** Social interaction, Tie formation, Tie maintenance, Loneliness, Built environment, Urban form, Health, Well-being, Activity space model, PPGIS

## Abstract

**Background:**

Social interaction is essential for health and well-being, given the growing public health concern of social isolation and loneliness. The role of the built environment in supporting social interaction has been widely studied. However, previous research has often treated social interaction as a single, undifferentiated category, although different types of interaction may serve distinct social functions and be influenced by different environmental factors. Moreover, most studies have focused primarily on residential neighborhood contexts. This study addresses these key gaps by distinguishing between two types of social interaction—tie formation and tie maintenance—and by examining built environment characteristics across broader, individualized multidimensional activity space models.

**Method:**

Using data from a Public Participatory GIS (PPGIS) survey (*n* = 386) in Turku, Finland, this study analyzed how residential urban form and built environment features relate to tie formation and tie maintenance. Built environment features were assessed using three activity space models: 500-meter home buffer, combined buffer around home and daily destinations, and individualized activity range spanning between home and destinations. Structural Equation Modeling was used to examine how these factors influence each type of social interaction and associated psychosocial outcomes.

**Results:**

Residing in urban areas was significantly associated with tie maintenance but not with tie formation. Walkability around the home supported both types of interaction, whereas parks and green spaces near daily destinations were positively associated with tie formation. A similar pattern was observed within individualized activity ranges, where park ratio predicted tie formation. These two types of social interaction influenced psychosocial outcomes through distinct pathways: tie formation had direct positive effects on health and well-being, while tie maintenance contributed indirectly through increased relationship satisfaction.

**Conclusion:**

The findings emphasize the importance of distinguishing between different types of social interaction and accounting for their unique spatial and functional drivers. Urban planning and public health efforts should consider how different aspects of the built environment foster both the formation and maintenance of social ties. Promoting environments that support diverse forms of social interaction is essential not only for enhancing health and well-being but also for reducing the risk of loneliness.

**Supplementary Information:**

The online version contains supplementary material available at 10.1186/s12942-026-00451-z.

## Background

Social isolation and loneliness have become a growing public health concern. Social isolation refers to an objective lack of social contacts, whereas loneliness reflects a subjective discrepancy between the actual and desired level of social connection [1]. Both of these constructs have been associated with adverse health consequences, such as elevated mortality comparable to smoking or chronic alcohol consumption [2], heightened psychological distress and poorer mental health outcomes [3], and lower levels of subjective well-being [4]. Given the recent trends of increasing social isolation and loneliness, which have been exacerbated by the COVID-19 pandemic [5], identifying environmental and social conditions that can alleviate these issues has become increasingly important. While both constructs are relevant, this study adopts a loneliness perspective and focuses on how built environment characteristics may help improve the subjective dimensions of social relationships.

Promoting positive social ties through social interaction is a key protective factor against loneliness [6–8]. Social relationships are furthermore a fundamental component of general health and well-being [9–11]. In particular, satisfying personal relationships, such as those with partners, family, and friends, are strong predictors of well-being, often outweighing the impact of income [12]. In addition, satisfaction with personal relationships plays a crucial role in how the surrounding environment influences well-being, acting as a key mediating factor in this association [13–15].

However, not all social ties serve the same function. Weak ties, often newly formed social relationships, facilitate access to new information and opportunities by connecting socially dissimilar individuals, whereas strong ties offer emotional support and companionship based on close, emotionally involved relationships [16, 17]. Social network frameworks conceptualize social life as a balance between two simultaneous and dynamic relational processes: tie formation and tie maintenance [18, 19]. In practice, tie formation generally involves the development of weak ties, creating new relationships that expand one’s network and access to novel resources, while tie maintenance usually involves the strengthening of existing strong ties, nurturing emotional support and stability within a network. Furthermore, previous research suggests that different dimensions of social ties, such as type, strength, and structure, can influence psychological and health outcomes differently [11, 20, 21]. These findings indicate that forming new connections and maintaining existing ones may play distinct, yet complementary roles in promoting health and well-being.

In this context, a substantial body of research has examined how specific features of the built environment shape social interaction. A review paper also highlights that built environments afford opportunities for social interaction, which can help prevent loneliness [22]. Commonly studied indicators include walkability, the availability of third places, green spaces, and parks.

Walkability is one of the important factors that are associated with greater social engagement [23–26]. It also promote social inclusion by reducing transportation disadvantages [27], and has been to shown to increase the frequency of meaning social interactions [28]. These findings resonate with the early urban theories of Jane Jacobs [29], who emphasized that walkability and land-use diversity are essential for fostering vibrant street life and diverse social interaction. Third places—informal gathering venues such as cafés, restaurants, and bars—are recognized for enabling spontaneous, repeated encounters outside the home and workplace [30–32]. These spaces also contribute to reducing loneliness [33] and promoting well-being [34]. Green spaces, such as forests and natural areas, have been linked to greater social cohesion and encourage positive encounters among neighbors [35–37]. Similarly, urban parks play a vital role in fostering social activities within neighborhoods [38, 39]. When located in the neighborhood within walking distance, parks also promote pedestrian activity, increasing the likelihood of spontaneous social interaction during daily routines [40], suggesting that parks may support social ties both by offering communal spaces and by encouraging active mobility that facilitates serendipitous engagement. Beyond these specific features, studies also suggest that living in compact urban environments promotes more social interaction by providing greater opportunities for encounters and engagement [14, 41].

Despite this growing body of literature, two key gaps remain. First, many empirical studies exploring built environment influences on social interaction have overlooked the distinction between tie formation and tie maintenance, and instead treated social interaction as a single, undifferentiated category [42]. Yet these two processes could serve different social functions and may be shaped by different environmental factors. Failing to distinguish between them may obscure how specific features of the built environment facilitate different types of social engagement and, in turn, how they relate to psychosocial outcomes, including subjective health and well-being. Second, much of the existing research has focused on neighborhood-level built environments around the home and, correspondingly, on social interaction occurring within the immediate residential area. However, Mouratidis [14] argues that studies should move beyond neighborhood-level perspectives to consider individuals’ overall social networks. Similarly, Jones and Pebley [43] demonstrate that focusing solely on residential neighborhoods captures only a fraction of the social and spatial environments individuals actually engage with in their daily lives. Furthermore, studies have shown that the associations between built environment characteristics and health outcomes can vary depending on the geographic scale or spatial context considered [44, 45], highlighting the importance of carefully defining spatial units in research.

To address these gaps, this study proposes a conceptual framework that distinguishes between two types of social interaction—tie formation and tie maintenance—reflecting their distinct relational functions. Furthermore, to move beyond the limited focus on the residential neighborhood, we incorporate activity space models that capture not only the area around the home but also daily destinations and the broader spatial ranges connecting them, thereby allowing for an assessment of built environment influences beyond the neighborhood level. Activity space approaches have been proposed and developed to represent a broader set of spatial contexts shaped by individuals’ daily routines [46–49]. These models contribute to a more nuanced understanding of how built environment features influence health outcomes [50–52], as well as social outcomes [48, 53]. In line with this perspective, our analysis focuses on general social activities involving interactions with others—such as friends, partners, or new acquaintances—beyond neighborhood-level social engagement. Using empirical data collected in Turku, Finland, we first compare how participation in these two types of social activities varies between urban and peri-urban residents, both in terms of likelihood and spatial distribution. Building on this, we apply Structural Equation Modeling (SEM) to examine how the location of residence and detailed built environment features, assessed through multidimensional activity space models, influence psychosocial outcomes through tie formation and maintenance.

In particular, we aim to address the following research questions (RQs) through this study:

RQ1: How do different residential urban form and built environment features influence two distinct types of social interaction—tie formation and tie maintenance—across multidimensional activity space models?

RQ2: Do tie formation and tie maintenance mediate the relationship between built environment features and psychosocial outcomes, specifically relationship satisfaction, subjective health, and subjective well-being?

## Method

### Study area and participants

This study uses survey data collected in Turku, a mid-sized city in Southwest Finland with a population of approximately 206,000 in 2024, which grew by 2% from the previous year, according to Statistics Finland [54]. The same statistics report an average age of 42 years among Turku resident, with an age distribution of 12% under 15 years, 68% between 15 and 64 years, and 20% aged 65 or older. In terms of demographic composition, 11% of residents were foreign citizens, while 17% had a foreign background.

To capture both spatial and non-spatial dimensions of individuals’ social activity patterns, a Public Participation GIS (PPGIS) survey was carried out in Turku between May and June 2023. PPGIS enables participants to mark places that are meaningful in their daily lives—such as home, work, study locations, and other frequently visited places—on an interactive digital map. This method provides valuable insight into how people experience and interact with their everyday environments [55]. A key feature of the PPGIS survey conducted in Turku was the collection of geolocated data on participants’ everyday places—including home, work, study, and childcare locations—as well as locations perceived to support their well-being across physical, mental, and social dimensions. In addition to mapping these locations, the survey included general rating questions related to participants’ subjective health and well-being.

For the well-being component, participants were asked to identify on the map all locations they considered beneficial to their physical, mental, and social well-being. The instruction was phrased as follows: “*Please mark on the map as many places as you can that you think support socializing and meeting with others*,* are important for you to recover from stress and helps you relax and you use for regular physical activities. You can mark the same places multiple times if you believe they are important for your well-being in different ways*.” The question did not impose a specific recall period but instead encouraged respondents to reflect on places they generally associate with well-being in their everyday lives. For each marked well-being location, respondents were subsequently asked to report how frequently they visit the location and which mode of transport they usually use to travel there. Furthermore, if the place was considered relevant to socializing and meeting with others (social well-being), an additional question was prompted: “*What do you typically do at this place?*” Participants could then choose from predefined options, including ‘*meet new people and make friends*’, ‘*attend social events or group activities*’, ‘*be around other people*’, ‘*meet with friends or family*’, ‘g*et support from other people*’, or select ‘*other*’ if none applied.

**Fig. 1:**
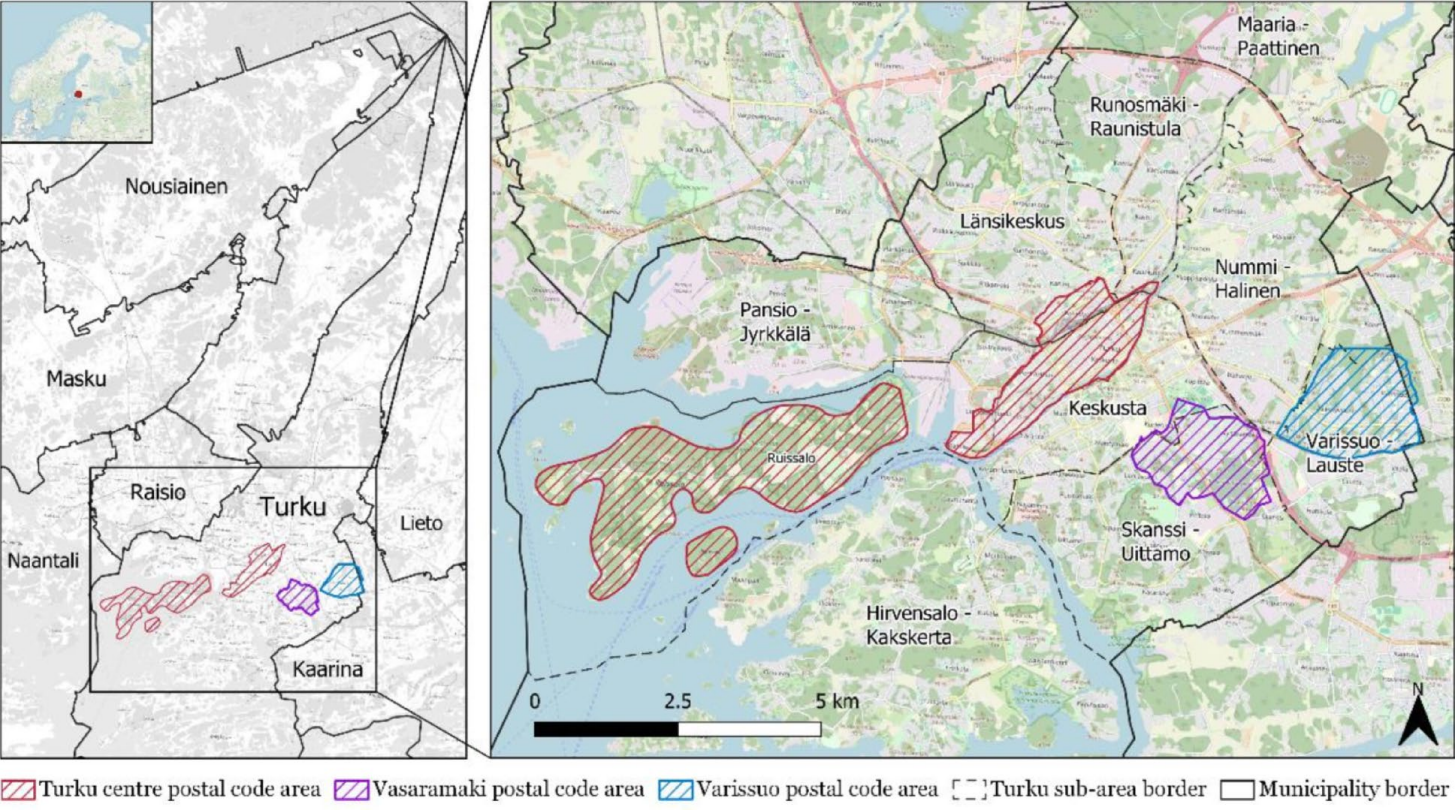
Data collection areas in Turku

A total of 5,000 invitation letters were distributed to randomly selected residents aged 15 to 85 across three postal code areas of Turku (Turku Centre, Vasaramäki, and Varissuo) chosen for their variation in demographic composition and built environment characteristics. No additional inclusion or exclusion criteria were applied. For analytical purposes, the Turku Centre was classified as an urban area, with the exception of the Ruissalo island area. Although Ruissalo shares the same postal code, it is geographically distinct and more appropriately categorized as peri-urban due to its lower density and land use characteristics. While not rural, such areas differ from the compact urban core and are better described as peri-urban zones, transitional spaces between urban and rural environments (see Fig. 1). The survey yielded 435 responses, resulting in an overall response rate of 8.7%. Prior to analysis, the data were cleaned to ensure quality and consistency. Incomplete responses lacking home location and duplicate markings were removed, resulting in a final dataset of 386 respondents. Table 1 presents the sociodemographic characteristics of respondents overall and across the three data collection areas. Turku Centre accounted for the largest share of responses, with a higher proportion of younger respondents aged 15–29. In contrast, the peri-urban areas of Vasaramäki and Varissuo had higher proportions of respondents living with children, reflecting differences in household composition across locations. The overall age composition of the sample shows that 79% of respondents were aged 15–64, and 21% were 65 or older (combining the 15–29 and 30–64 age groups). In comparison, Turku’s citywide population statistics report that 77% of residents are aged 15–64 and 23% are 65 or older, excluding those under 15. Since the survey targeted individuals aged 15 and above, the comparison suggests no major sampling bias in terms of age distribution.

**Table 1:** Sociodemographic characteristics of respondents

Variable	Total (*N* = 386)	Turku Centre (*N* = 283)	Vasaramäki (*N* = 42)	Varissuo (*N* = 61)
Gender (%)				
Female	56%	55%	60%	61%
Age group (%)				
15–29 years old	25%	30%	10%	15%
30–64 years old	54%	51%	78%	54%
65 years or older	21%	20%	12%	31%
Household type (%)				
Living with partner	56%	54%	69%	57%
Living with children	14%	10%	29%	21%
Household income				
< 2000 EUR/month	27%	29%	17%	26%
2000–3999 EUR/month	34%	31%	36%	46%
4000–5999 EUR/month	24%	24%	31%	18%
6000–7999 EUR/month	6%	6%	10%	2%
> 8000 EUR/month	9%	10%	7%	8%

### Social interaction variables

Using the collected data, social interactions were classified into two distinct types—tie formation and tie maintenance—based on respondents’ reported social activity type for each location. Specifically, each social activity was categorized as tie formation if the respondent indicated it involved ‘*meet new people and make friends’*,* ‘attend social events or group activities’*, or *‘be around other people’*. Conversely, activities were classified as tie maintenance if they were reported as involving *‘meet with friends or family’*. While the present dataset does not directly measure the strength of each individual social tie, this classification aims to distinguish between interactions that primarily serve the purpose of initiating new or casual ties (tie formation) and those aimed at maintaining established, emotionally meaningful relationships (tie maintenance). Meeting with friends or family was classified as tie maintenance, as it involves reinforcing existing close relationships. In contrast, meeting new people and making friends, attending social events or group activities, and being around other people were classified as tie formation, as these activities typically involve engagement with individuals beyond one’s established. Responses marked as *‘get support from other people’* or *‘other’* were excluded from this classification, as their interpretation in terms of tie formation or maintenance was ambiguous. Each respondent could only select one main activity type per mapped location, ensuring no overlap in categorization. In total, 137 activity points were categorized as tie formation places, and 178 as tie maintenance places.

At the respondent level, tie formation and tie maintenance were treated as binary variables: individuals were coded as having engaged (1) or not engaged (0) in each type of social interaction, based on whether they had marked at least one location corresponding to each category. Some respondents engaged in both types of social interaction at different locations, while others engaged in only one type or in neither. This binary approach was adopted instead of using count variables, as the focus was on the occurrence of each interaction type rather than its frequency or intensity.

### Psychosocial variables

This study focused on three key psychosocial outcomes that are theoretically influenced by social interaction: *Relationship satisfaction*, *Subjective health*, and *Subjective well-being*.

*Relationship satisfaction* was measured using a single item from the PPGIS survey, which asked participants to rate their satisfaction with personal relationships on a five-point scale (1 = *very dissatisfied* to 5 = *very satisfied*). The wording of this item follows that used in widely applied international surveys such as those by Eurostat [56].

*Subjective health* was modeled as a latent construct composed of three self-reported indicators from the PPGIS survey: (i) general health, (ii) physical health, and (iii) mental health. In line with the World Health Organization (WHO) definition, health encompasses physical, mental, and social dimensions [57]. However, as the term ‘social health’ is not commonly used in self-assessment surveys [58], we relied on general, physical, and mental health ratings to capture a comprehensive view of subjective health and reduce potential measurement error. Participants evaluated each of the three health dimensions on a five-point scale from 1 (*very poor*) to 5 (*very good*).

*Subjective well-being* was also modeled as a latent construct, using two items from the PPGIS survey: (i) happiness and (ii) quality of life. This operationalization aligns with OECD guidelines, which define subjective well-being in terms of three components: affect (emotional states or feelings), life evaluation (reflective assessment of one’s life), and eudaimonia (sense of meaning and purpose in life) [59]. In this study, happiness captures the affective dimension, while quality of life reflects life evaluation. Although the eudaimonic component could not be assessed due to data limitations, these two items provide a meaningful approximation of subjective well-being. Participants rated each item on a five-point scale ranging from 1 (*very poor*) to 5 (*very good*).

To confirm the validity of two latent constructs—*Subjective health* and *Subjective well-being*—and ensure that the selected items accurately represented the underlying dimensions, a confirmatory factor analysis (CFA) was conducted (Table 2). This and all subsequent analyses were performed in R (version 4.4.2) using RStudio, with model estimation carried out using the *lavaan* package. The results show that all standardized factor loadings exceeded 0.70, indicating satisfactory internal consistency [60]. The adequacy of the measurement model was evaluated using standard fit indices, including the Comparative Fit Index (CFI), Tucker–Lewis Index (TLI), and Standardized Root Mean Square Residual (SRMR). Following several guidelines [61, 62], we regarded CFI and TLI ≥ 0.95 as indicative of good fit, values between 0.90 and 0.95 as acceptable, and SRMR ≤ 0.08 as acceptable.

The overall model demonstrated a mixed fit to the data, with CFI = 0.901 and TLI = 0.833, and SRMR = 0.099. While the CFI reached the threshold for acceptable fit, the TLI and SRMR fell slightly below conventional cutoffs, possibly due to the limited number of items per construct. Nonetheless, the model demonstrated sufficient internal consistency and theoretical coherence to justify its use in the subsequent analyses.

**Table 2:** Confirmatory factor analysis results for psychosocial variables

Variable	Standardized coefficient
*Subjective health*	
SH1: General health	0.901
SH2: Physical health	0.819
SH3: Mental health	0.701
*Subjective well-being*	
SW1: Happiness	0.905
SW2: Quality of life	0.869

Model fit measures: CFI = 0.901; TLI = 0.833; SRMR = 0.099

### Built environment variables in a multidimensional activity space model

To assess the built environment variables relevant to social interaction, four key variables were calculated: walkability index, third place density, park ratio, and green space ratio. All indicators were derived from OpenStreetMap (OSM) data and computed within defined activity spaces based on participants’ homes and activity locations.

The walkability index was calculated as the sum of standardized scores (z-scores) of three core components, following the methodology proposed by Frank et al. [63]: residential density, intersection density, and land use mix entropy. Retail floor area, a commonly used fourth component in walkability indices was excluded in this study due to limited data availability and to avoid conceptual overlap with third places density, which was included as a distinct variable. Residential density was calculated as the proportion of residential land use within each respondent’s individualized activity space buffer. Using OSM land use data, polygons tagged as ‘residential’ were extracted and intersected with each buffer. The total residential land area within each buffer was then divided by the total buffer area to produce a continuous density measure ranging from 0 to 1. Intersection density was defined as the number of unique pedestrian street intersections per square kilometer. Using OSM road data, the start and end points of pedestrian segments were snapped and deduplicated to identify intersections. This method captures both three-way and four-way intersections and reflects the degree of pedestrian connectivity within each buffer. The count of unique points was divided by buffer area to compute the density. Land use mix was assessed using the Shannon entropy index based on the relative area of four key land use categories within each buffer: residential, commercial, retail, and industrial. The entropy score was calculated using the following formula:


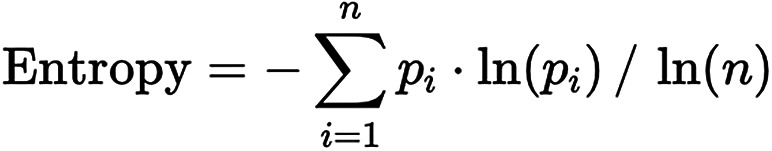


where *pi* is the proportion of land area belonging to land use category *i*, and *n* is the total number of land use categories considered.

All three walkability components were standardized (z-scores) and then summed to produce the final walkability score for each respondent. Higher scores indicate more walkable and functionally diverse urban environments. While this composite index captures key structural features of walkability, it does not include qualitative features of the pedestrian environment, such as sidewalk infrastructure, street furnitures, or perceived safety, due to data limitations.

Third places density was calculated as the number of cafés, restaurants, and bars within each spatial unit, normalized by area. These venues serve as informal settings for socialization beyond home and work [30]. The calculation was based on OSM POI data, filtered by relevant categories (cafe, bar, restaurant), with density expressed as POIs per km².

Park ratio was calculated as the proportion of park area within each buffer, using OSM land-use data where polygons classified as park were extracted and intersected with the buffer. Similarly, green space ratio was defined as the percentage of natural green areas—such as forests, grasslands, and meadows—within the buffer, based on polygons tagged as forest, grassland, or meadow. While parks and green spaces are often grouped together, they serve different functions: green spaces are more closely associated with preserved nature, while parks represent explicitly managed urban vegetation [64]. Accordingly, this study distinguished between these environmental features.

**Fig. 2:**
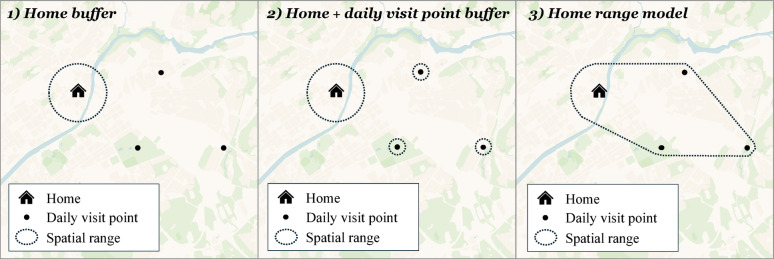
Illustration of three activity space models

To account for individual differences in spatial behavior and environmental exposure, the built environment variables described above were calculated using three distinct activity space models (Fig. 2). This multidimensional spatial framework enables a more precise assessment of how environmental contexts at varying spatial scales relate to social activity patterns.

The first model, *home buffer*, applied a 500 m circular buffer around each participant’s home location to represent their immediate residential environment. This buffer size is widely used in urban studies as a proxy for walkable neighborhoods [49, 65]. The second model, *home + daily visit point buffer*, combined a 500 m buffer around the home with a 140 m buffer around places that participants reported visiting more than once per week, such as workplaces, schools, childcare facilities, and gyms. The 140 m distance reflects the typical spatial extent of activity clusters identified in prior studies [49, 52]. The third model, *home range model* introduced by Hasanzadeh et al. [49], extended the second model by incorporating not only the areas around the home and frequently visited locations but also the broader individualized activity range encompassing them. For each participant, this model captured an activity space defined by a convex hull that connects the home location with all mapped places visited at least once per week. To reduce the influence of spatial outliers, a distance threshold from the home was applied using the Jenks natural breaks classification method, with the optimal number of classes determined based on the goodness of variance fit criterion [66].

By employing these three activity space models, this study assesses not only residential built environment conditions but also those encountered across participants’ broader daily activity ranges, thereby offering deeper insights into how different environmental contexts support social interaction.

OSM data was used to derive built environment indicators due to its comprehensive spatial coverage—including land use polygons, road networks, and points of interest (POIs)—which can be efficiently integrated into activity space models. In addition, it supports cross-country methodological consistency, enhancing the reproducibility and comparability of the study. While OSM is a crowd-sourced platform, its accuracy and completeness are reported to be high particularly in European contexts [67]. To assess its validity in our study area, we conducted a basic comparison of selected OSM-based indicators with administrative datasets such as CORINE and NDVI. The results showed that the OSM indicators aligned well with the reference datasets, supporting the use of OSM in this context (see Appendix A).

### Residential urban form (urban or peri-urban residents)

**Fig. 3:**
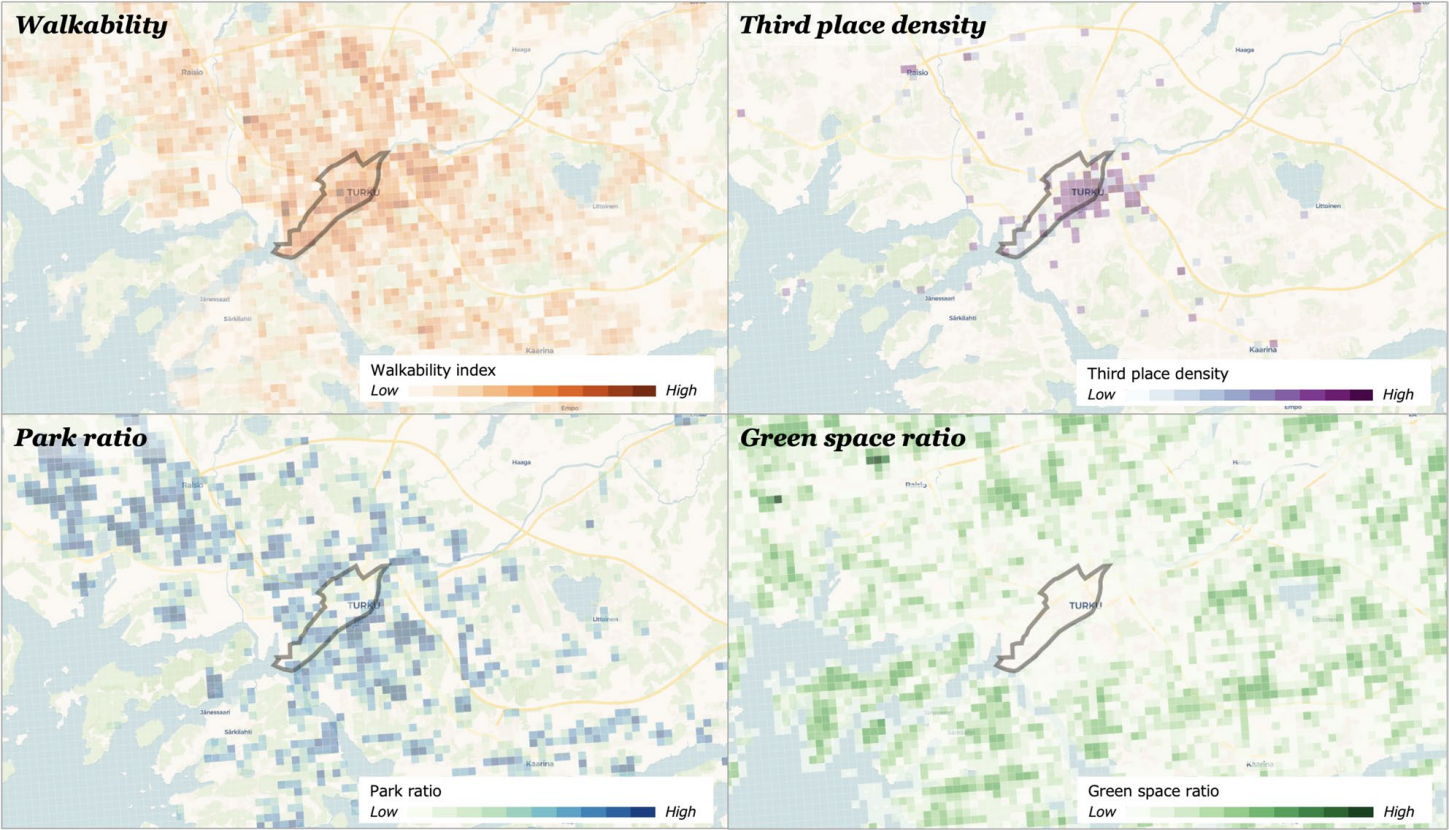
Boundary of urban setting (grey polygon) and spatial distribution of built environment

This study categorized respondents’ residential urban form into two groups based on postal code areas collected through the survey. For the purpose of comparison, Turku Centre (excluding Ruissalo island area) was classified as an urban setting, while Vasaramäki, Varissuo, and Ruissalo island area were grouped as peri-urban settings, serving as transitional zones between urban and rural settings. Accordingly, respondents living in Turku Centre were referred to as urban residents (274 samples), and those in the other areas as peri-urban residents (112 samples). Figure 3 illustrates the boundary of the urban setting and the spatial distribution of each built environment feature, aggregated across 250 × 250 m grid cells for visualization.

Using this categorization, Table 3 presents descriptive comparisons between urban and peri-urban residents using a *t*-test. The table includes standardized scores for the psychosocial variables derived from the previously described factor analysis, along with measures of built environment characteristics calculated within a 500 m buffer around each respondent’s home. Additionally, key sociodemographic variables such as age, gender, household composition, and income are reported to provide context for interpreting the observed differences. Participation rate in tie formation and tie maintenance was analyzed separately, and the results are presented in the Results section, specifically in the subsection on comparison of social interaction patterns between urban and peri-urban residents.

As shown in Table 3, there were no statistically significant differences in psychosocial variables between urban and peri-urban residents, although urban residents reported slightly higher levels of relationship satisfaction, subjective health, and well-being. In contrast, built environment characteristics within the 500 m home buffer showed substantial and statistically significant differences. Urban residents lived in areas with higher walkability, greater density of third places, and more park coverage, whereas peri-urban residents were surrounded by a significantly higher proportion of green space such as forested areas. Regarding sociodemographic characteristics, peri-urban residents were on average older and more likely to live with children compared to their urban counterparts. No significant differences were observed in gender distribution, cohabitation with a partner, or household income between the two groups.

**Table 3:** Descriptive comparison of variables between urban and peri-urban residents

Variable	Urban Residents (*N* = 274)		Peri-urban Residents (*N* = 112)		t-test
	Mean	SD	Mean	SD	
*Psychosocial variables (standardized)*					
Relationship Satisfaction	0.07	(0.99)	−0.03	(1.02)	
Subjective Health	0.07	(0.99)	−0.06	(0.99)	
Subjective Well-being	0.09	(1.01)	−0.07	(0.98)	
*Built environment (500 m buffer from home)*					
Walkability	0.67	(0.09)	0.40	(0.11)	***
Third places density	39.69	(38.96)	2.29	(10.68)	***
Park ratio	0.11	(0.05)	0.08	(0.08)	***
Green space ratio	0.03	(0.02)	0.24	(0.12)	***
*Sociodemographic variables*					
Age (continuous variable)	43.82	(19.05)	50.77	(16.67)	***
Female (binary; 1 = female)	0.54	(0.50)	0.61	(0.49)	
Living with partner (binary; 1 = yes)	0.54	(0.50)	0.62	(0.49)	
Household with children (binary; 1 = yes)	0.10	(0.30)	0.23	(0.42)	**
Household income (mid-point value; Euro/month)	3045.26	(1389.16)	3182.29	(1267.24)	

^a^: *p* < 0.10, *: *p* < 0.05, **: *p* < 0.01, ***: *p* < 0.001

### Analysis approach

This section presents the analytical procedures in alignment with the study’s two research questions: (RQ1) How do different residential urban form and built environment features influence two distinct types of social interaction across multidimensional activity space models? (RQ2): Do tie formation and tie maintenance mediate the relationship between built environment features and psychosocial outcomes?

To investigate how residential urban form and built environment characteristics relate to social interaction, this study first conducts a preliminary analysis comparing two types of social activity—tie formation and tie maintenance—between urban and peri-urban residents. Specifically, participation rates in each type of social activity are compared across the two residential groups. In addition, for respondents who reported engaging in social activities, the distance from home to the reported activity locations is examined. This analysis contributes to RQ1 by clarifying how residential context is associated not only with the likelihood of participating in social interaction but also with the spatial patterns of those interactions.

Building on this, the study applies SEM to examine the pathways linking residential urban form and built environment characteristics to psychosocial outcomes, mediated by two distinct types of social interaction: tie formation and tie maintenance. These two social interaction variables are specified as mediators, predicted either by a binary residential urban form—urban versus peri-urban (model 1) or by built environment features calculated through three activity space models: a 500 m buffer around the home (model 2.1: *home buffer*), a combined buffer around the home and frequently visited locations (model 2.2: *home + daily visit point buffer*), and an individualized activity range spanning between home and daily destinations (model 2.3: *home range model*). Built environment variables—walkability, third places density, park ratio, and green space ratio—are each calculated within these spatial frameworks. This modeling approach addresses RQ1 by enabling a comparison of how different built environment features influence tie formation and tie maintenance across multiple spatial exposure models. It also addresses RQ2 by evaluating the extent to which the effects of the built environment on psychosocial outcomes are mediated by these two types of social interaction.

**Fig. 4:**
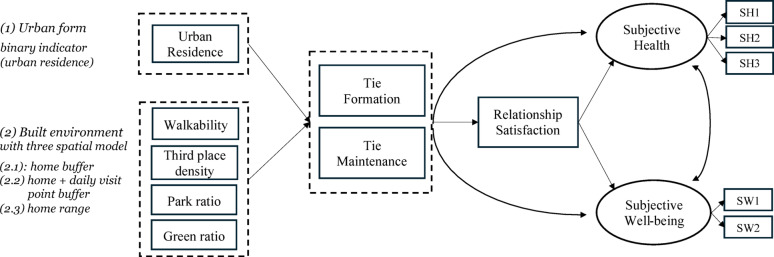
Overall conceptual path structure in all four models

Figure 4 presents the overall conceptual path structure in all four models. Environmental variables—including residential urban form and built environment features—are hypothesized to influence tie formation and tie maintenance, which in turn affect relationship satisfaction, subjective health and well-being. Based on previous research [13–15], relationship satisfaction is modeled as a key mediator, predicting subjective health and well-being. Subjective health and subjective well-being are included in the SEM as latent constructs, defined through confirmatory factor analysis based on multiple survey items. A correlation is also specified between subjective health and well-being based on precious findings [68]. In line with existing research [69], which highlight the importance of sociodemographic factors in shaping social interaction alongside built environmental conditions, we included age, gender, household type, and household income as control variables for all model variables. For clarity, these controls are not shown in the conceptual diagram.

SEM estimation was conducted using maximum likelihood in R, as described in the CFA section. Model fit was evaluated using standard indices (CFI, TLI, and SRMR), with the same thresholds applied as in the CFA: CFI and TLI ≥ 0.95 were interpreted as indicating good fit, values between 0.90 and 0.95 as acceptable, and SRMR ≤ 0.08 as acceptable.

## Results

### Comparison of social interaction patterns between urban and peri-urban residents

**Fig. 5:**
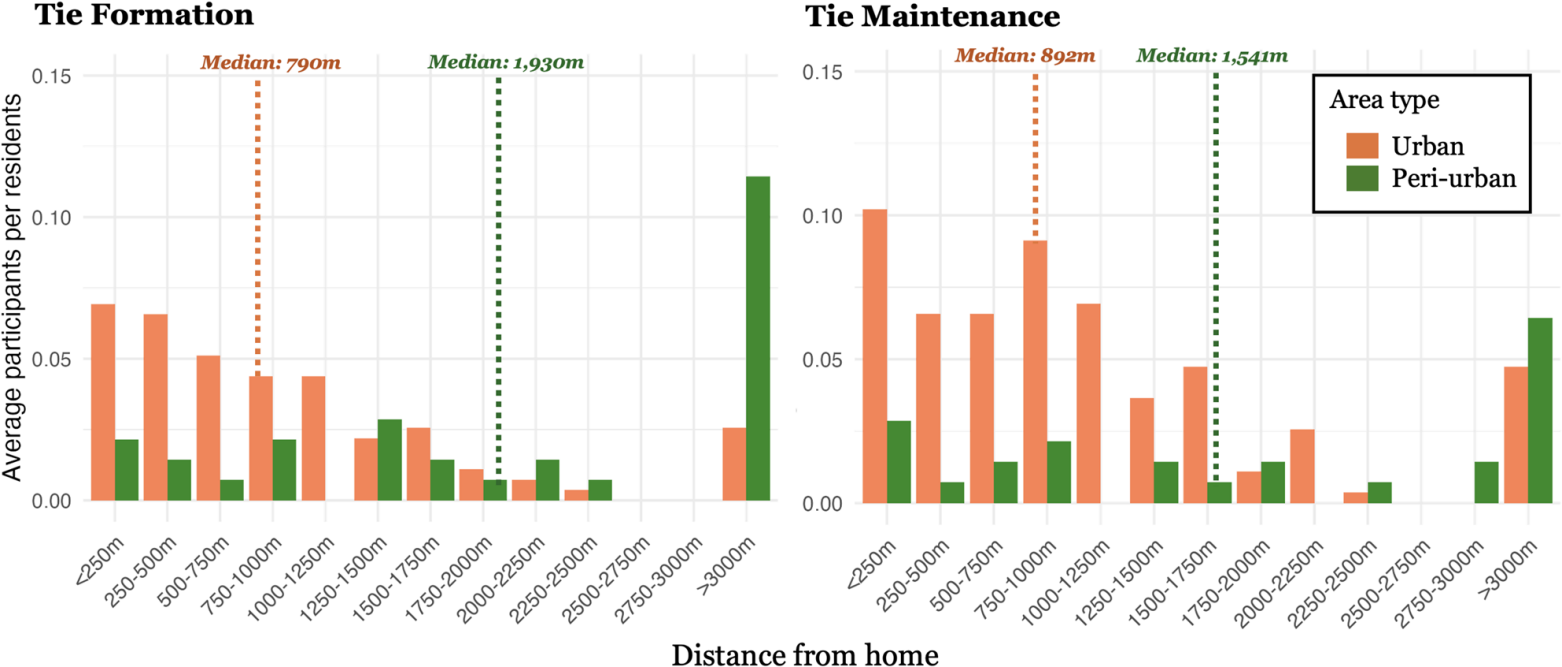
Distribution of distance from home to each social activity place

As a preliminary step adressing RQ1, this section examined how residential urban form is associated with participation in social activities by comparing urban and peri-urban residents in terms of two distinct types of social interaction: tie formation and tie maintenance. Table 4 presents participation rates and the median distances from home to the reported social activity locations (excluding non-participants), while Fig. 5 illustrates the detailed distribution of these distances. In Table 4, a respondent was counted as a participant in tie formation, or tie maintenance, if they reported at least one social activity location related to that respective type. In Fig. 5, the percentages on the vertical axis represent the proportion of participants in each distance category, calculated separately for tie formation and tie maintenance, and normalized by the total number of participants in the corresponding urban or peri-urban group.

To assess statistical differences between urban and peri-urban residents, Fisher’s exact test was used for comparing participation rates in each social activity type, given their categorical nature. For comparing the distances from home to reported activity locations, the Wilcoxon rank-sum test was applied, as the distance data are peri-normally distributed.

Regarding tie formation, the overall participation rate did not differ significantly between urban residents (27.4%) and peri-urban residents (22.3%), according to Fisher’s exact test. However, a substantial difference was observed in the spatial distribution of these activities: urban residents engaged in tie formation activities significantly closer to home (median = 790 m) than peri-urban residents (median = 1,930 m, *p* < 0.01).

In contrast, tie maintenance showed clearer spatial and behavioral differences. The participation rate among urban residents (36.9%) was more than twice that of peri-urban residents (19.6%), with the difference approaching statistical significance (*p* < 0.10). Moreover, urban residents conducted tie maintenance activities significantly closer to home (median = 892 m) than their peri-urban counterparts (median = 1,541 m, *p* < 0.05).

The results indicate distinct patterns between urban and peri-urban residents regarding social activity participation. While the participation rate for tie formation did not significantly differ between the two groups, urban residents tended to engage in these activities at significantly shorter distances from home. In contrast, tie maintenance showed clearer differences, with urban residents not only participating at a higher rate than peri-urban residents but also conducting these activities significantly closer to their homes. These findings highlighted spatial differences in social interaction patterns across urban and peri-urban contexts.

**Table 4:** Comparison of social activity participation patterns

Variable	Urban residents (*N* = 274)	Peri-urban residents (*N* = 112)	*p*-value
*Tie Formation*			
Participation rate	27.4%	22.3%	
Distance from home (median)	790 m	1,930 m	**
*Tie Maintenance*			
Participation rate	36.9%	19.6%	^a^
Distance from home (median)	892 m	1,541 m	*

For calculating p-value, fisher’s exact test was applied for participation rate (categorical), and wilcoxon rank-sum test was applied for distance (non-normal distribution)

^a^: *p* < 0.10, *: *p* < 0.05, **: *p* < 0.01, ***: *p* < 0.001

### Structural equation modeling

This section presents the results of the SEM analyses conducted to examine how residential urban form and built environment characteristics influence psychosocial outcomes through two types of social interaction, addressing both research questions (RQ1 and RQ2). While the environmental predictors varied across models, the structure of the pathways from social interaction to psychosocial outcomes remained consistent. The following subsections therefore first present the common effects of tie formation and tie maintenance on psychosocial outcomes, followed by a comparison of environmental predictors across the four models.

1. ***SEM: Effects of social interaction on psychosocial outcomes***

Across all four SEM models, consistent patterns were observed in the relationships between social interaction and psychosocial outcomes. To clarify these pathways independently of environmental predictors, a simplified structural equation model was estimated, excluding built environment characteristics and urban form variables.

Table 5 presents the standardized direct effects of tie formation and tie maintenance on relationship satisfaction, subjective health, and subjective well-being, while also incorporating the direct effects of relationship satisfaction on health and well-being. This model isolated the core psychosocial pathways within the broader framework and demonstrated acceptable overall fit (CFI = 0.955, TLI = 0.913, SRMR = 0.057), all of which fall within the acceptable range based on defined criteria (CFI/TLI ≥ 0.90; SRMR ≤ 0.08).

After controlling for sociodemographic variables, tie formation was found to have a statistically significant positive association with both subjective health (β = 0.113, *p* < 0.05) and subjective well-being (β = 0.091, *p* < 0.05), but not with relationship satisfaction. This suggests that engaging in activities that involve meeting new people or expanding one’s social network may contribute more directly to broader aspects of psychosocial functioning.

By contrast, tie maintenance was significantly associated with relationship satisfaction　(β = 0.120, *p* < 0.05), indicating that sustaining existing social ties plays an important role in enhancing individuals’ satisfaction with their personal relationships. Furthermore, relationship satisfaction consistently emerged as a strong predictor of both subjective health (β = 0.549, *p* < 0.001) and subjective well-being (β = 0.788, *p* < 0.001). The standardized path coefficients for these associations were consistently high, highlighting the central role of close relationships in supporting both health and well-being. In addition, subjective health and well-being were positively and significantly correlated, suggesting that these two outcomes are closely interrelated.

Some sociodemographic factors also showed notable associations: older age was linked to lower subjective health, while women reported higher relationship satisfaction. Also, higher income was positively associated with relationship satisfaction and subjective well-being, highlighting the continued role of individual characteristics in shaping psychosocial outcomes.

In addition to direct effects, indirect effects via relationship satisfaction were also tested and are presented in Table 6. Tie maintenance exhibited significant indirect effects on both subjective health (β = 0.066, *p* < 0.05) and subjective well-being (β = 0.095, *p* < 0.05), mediated by relationship satisfaction. This suggests that tie maintenance influences psychosocial outcomes primarily through its effect on close relationship satisfaction. In contrast, the indirect effects of tie formation on either outcome were not statistically significant.

Taken together, the findings show that tie formation and tie maintenance have distinct associations with psychosocial outcomes. Specifically, maintaining existing ties was associated with higher relationship satisfaction, which indirectly supports both subjective health and well-being. In contrast, forming new ties was directly associated with subjective health and well-being.

**Table 5:** Standardized effects of social interaction on psychosocial outcomes (direct effects)

	Dependent variables		
	Relationship satisfaction	Subjective health	Subjective well-being
*Social interaction*			
Tie formation	0.030	0.113*	0.091*
Tie maintenance	0.120*	0.080	0.013
*Relationship satisfaction*	-	0.549***	0.788***
*Control variables*			
Age	−0.077	−0.172*	−0.063
Female	0.133*	−0.062	−0.053
Living with partner	0.010	0.058	0.039
Household with children	−0.103	−0.002	−0.009
Household income	0.150*	0.076	0.119 *

Covariances: Subjective health and subjective well-being (Standardized estimate = 0.270***)

Model fit measures: CFI = 0.955; TLI = 0.913; SRMR = 0.057

^a^: *p* < 0.10, *: *p* < 0.05, **: *p* < 0.01, ***: *p* < 0.001

**Table 6:** Standardized effects of social interaction on psychosocial outcomes via relationship satisfaction (indirect effects)

	Dependent variables	
	Subjective health	Subjective well-being
*Social interaction (via relationship satisfaction)*		
Tie formation	0.016	0.024
Tie maintenance	0.066*	0.095*

Model fit measures: CFI = 0.955; TLI = 0.913; SRMR = 0.057

^a^: *p* < 0.10, *: *p* < 0.05, **: *p* < 0.01, ***: *p* < 0.001

(2) ***SEM: Predictors of social interaction across four models***

Building upon the consistent effects of social interaction on psychosocial outcomes, this section examines the environmental predictors of tie formation and tie maintenance across four models.

Table 7 summarizes the standardized path coefficients from the SEM assessing how residential urban form and built environment characteristics influenced social interaction. Model (1) included urban residence as a binary indicator of urban form, while Models (2.1), (2.2), and (2.3) applied multidimensional activity space approaches using built environment variables measured within different geographic extents: (2.1) a 500 m buffer around the home (*home buffer*), (2.2) a combined buffer around the home and frequently visited locations (*home + daily visit point buffer*), and (2.3) an individualized activity range spanning between home and daily destinations (*home range model*). All models controlled for individual sociodemographic variables, including age, gender, household type, and household income. In addition, covariances were specified between key built environment predictors based on observed correlations: a positive correlation between walkability and third places density, and a negative correlation between walkability and green space ratio. All four SEM models demonstrated overall acceptable fit based on standard criteria. Specifically, CFI values were all above the 0.90 threshold, and TLI values were generally close to or above 0.90, indicating acceptable model structure, although Models (2.1) to (2.3) slightly exceeded the conventional SRMR cutoff of 0.08.

In terms of residential urban form, Model (1) revealed that living in an urban area was significantly associated with higher levels of tie maintenance (β = 0.145, *p* < 0.01), whereas its effect on tie formation was nonsignificant. This suggested that urban environments may better support the continuation of existing relationships rather than the initiation of new ones.

Moving beyond the binary classification of urban residence, the three models incorporating spatially defined built environment characteristics offered more detailed insights. In Model (2.1): *home buffer*, walkability was positively associated with tie maintenance (β = 0.194, *p* < 0.01) and marginally associated with tie formation (β = 0.119, *p* < 0.10). In Model (2.2): *home + daily visit point buffer*, park ratio showed a significant positive association with tie formation (*β* = 0.127, *p* < 0.05), and green space ratio was marginally significant (*β* = 0.180, *p* < 0.10). Finally, in Model (2.3): *home range model*, park ratio again emerged as a significant positive predictor of tie formation (β = 0.111, *p* < 0.05), and walkability was marginally associated with tie maintenance (β = 0.202, *p* < 0.10).

To assess the robustness of these findings, we also tested alternative buffer thresholds (100 m, 200 m, 300 m) around daily visited destinations, in addition to the original 140 m threshold (see Appendix B). In Model (2.2), the results remained broadly consistent at 200 m and 300 m, with positive associations between park/green space exposure and tie formation generally preserved. In Model (2.3), the association between park ratio and tie formation remained stable across all distances, whereas the marginal association between walkability and tie maintenance was not observed at other thresholds, suggesting limited robustness.

Several sociodemographic controls also showed consistent patterns: women were more likely to participate in both interactions, and older age was negatively associated with tie maintenance. Other factors, including cohabitation, children, and income, showed no significant associations.

Overall, the findings indicated that residing in an urban area is associated with the maintenance of existing relationships, while the formation of new ties is more strongly influenced by specific environmental features. In particular, a walkable environment around the home was associated with both tie maintenance and formation. Furthermore, when exposure to environments around frequently visited destinations was considered, greater access to parks and green spaces was associated with higher levels of tie formation. This pattern was similarly observed when the spatial range between home and daily destinations was incorporated, where park exposure remained a consistent predictor of tie formation.

**Table 7:** Standardized effects of urban form/built environments on social interaction

	Model (1) Urban residence	Model (2.1) Home buffer	Model (2.2) Home + daily visit point buffer	Model (2.3) Home range model
***Path to ‘Tie formation*** **’**				
*Residential urban form*				
Urban residents	0.055	-	-	-
*Built environment feature*				
Walkability	-	0.119 ^a^	0.172	0.092
Third places density	-	0.028	0.006	−0.025
Park ratio	-	0.010	0.127*	0.111*
Green space ratio	-	0.063	0.180 ^a^	0.140
*Control variables*				
Age	0.014	0.007	−0.001	−0.025
Female	0.155**	0.147**	0.149**	0.149**
Living with partner	−0.083	−0.093	−0.094	−0.090
Household with children	0.048	0.058	0.050	0.029
Household income	−0.012	0.001	0.001	0.003
***Path to*** ***‘Tie maintenance’***				
*Residential urban form*				
Urban residents	0.145**	-	-	-
*Built environment feature*				
Walkability	-	0.194**	0.115	0.202 ^a^
Third places density	-	−0.087	−0.084	−0.123
Park ratio	-	0.056	0.088	0.013
Green space ratio	-	0.027	−0.077	−0.022
*Control variables*				
Age	−0.232**	−0.235**	−0.097 ^a^	−0.100 ^a^
Female	0.123*	0.121*	0.109 *	0.105 ^a^
Living with partner	0.028	0.027	0.050	0.038
Household with children	−0.008	−0.012	0.014	0.019
Household income	0.046	0.048	−0.023	−0.028

Standardized coefficients (Std. All) are reported

Model fit measures: (1): CFI = 0.946; TLI = 0.892; SRMR = 0.056; (2.1): CFI = 0.959; TLI = 0.941; SRMR = 0.087; (2.2): CFI = 0.942; TLI = 0.908; SRMR = 0.083; (2.3): CFI = 0.938; TLI = 0.901; SRMR = 0.087

Correlations between walkability and third places density ((2.1): *r* = 0.499; (2.2): *r* = 0.431; (2.3): *r* = 0.493)) and

between walkability and green space ratio ((2.1): *r* = −0.689; (2.2): *r* = −0.748; (2.3): *r* = −0.724)) are included

^a^: *p* < 0.10, *: *p* < 0.05, **: *p* < 0.01, ***: *p* < 0.001

## Discussion

### Environmental influences on social interaction

Findings from this study highlight the importance of differentiating between types of social interaction when examining environmental influences. Regarding the first research question (RQ1)—how different residential urban form and built environment features influence two distinct types of social interaction—the results indicate that tie formation and tie maintenance are shaped by different spatial and environmental factors.

First, urban residence was positively associated with tie maintenance, but not with tie formation. This suggests that compact urban settings may better support the continuation of existing social ties but are not sufficient in themselves to foster new connections. The preliminary analysis revealed that urban residents tend to engage in both types of social activity significantly closer to home. This spatial pattern implies that urban environments offer more proximate opportunities for social interaction, potentially lowering the barriers to participation—especially for maintaining existing relationships. These findings align with previous research [14], which indicates that residents of compact neighborhoods report greater satisfaction with their personal relationships partly due to more opportunities to sustain larger networks of close ties.

Beyond residential urban form, built environment characteristics showed more nuanced patterns. Walkability around the home was positively associated with both tie formation and tie maintenance, underscoring the social value of pedestrian-friendly neighborhoods. This finding is consistent with earlier research [23–26] that links walkability to social capital, but this study adds depth by demonstrating that walkability supports social interactions both for the formation of new ties and the maintenance of existing ones.

Furthermore, when spatial exposure was extended to include daily destinations, greater access to parks and green spaces around these locations was positively associated with tie formation. This suggests that not only the residential environment but also the environmental contexts encountered during routine daily activities play an important role in fostering new social connections. A similar pattern was observed when the spatial range between home and daily destinations was incorporated through individualized activity space models, with park exposure consistently predicting tie formation. These findings imply that green environments—whether near key destinations or across broader activity ranges—may encourage non-mandatory activities, such as spontaneous detours or extended trips, which in turn create opportunities for new social engagement.

These findings emphasize the importance of considering broader spatial exposure models. Not only residential neighborhoods, but also individualized activity ranges serve as social settings where both tie formation and maintenance can occur. This supports recent research suggesting that the choice of activity space model influences the observed associations between the built environment and behavioral outcomes [52, 70]. In sum, built environment features—especially walkability and access to green environments—play distinct yet complementary roles in shaping social interaction, depending on spatial context and type of tie.

### Social interaction as a mediator to psychosocial outcomes

The second research question (RQ2) asked whether tie formation and tie maintenance mediate the relationship between the built environment and psychosocial outcomes. The results demonstrate that these two types of social interaction serve as distinct mediators, each following different pathways.

Specifically, tie formation was directly associated with both subjective health and well-being. This suggests that forming new social ties may enhance individuals’ mental and emotional functioning by offering access to new experiences, social novelty, or a broader sense of connectedness. Such effects appear to operate independently of close personal relationships, offering more general psychosocial benefits.

In contrast, tie maintenance was not directly associated with health or well-being but was significantly associated with relationship satisfaction, which in turn strongly predicted both subjective health and well-being. This suggests an indirect pathway: maintaining strong ties fosters emotional security and perceived social support, which then enhance psychosocial outcomes. These findings are consistent with prior research [11, 20, 21], emphasizing the central role of strong, enduring ties in supporting psychological well-being through improved relationship quality.

Overall, this divergence in pathways illustrates that tie formation and tie maintenance each serve unique psychosocial functions. While newly formed ties may provide stimulation and expansion of social networks, maintained ties offer stability and deeper support. These results emphasize the need to conceptualize social interaction as a multifaceted process, where both types of relationships contribute to health and well-being in complementary but distinct ways.

Taken together, these findings provide a comprehensive view of how different aspects of the built environment influence distinct types of social interaction, and how these, in turn, relate to psychosocial outcomes. Specifically, walkable residential environments were found to support both the formation of new ties and the maintenance of existing ones, while green spaces and parks, particularly those located near daily activities, were more strongly associated with tie formation. In contrast, urban residence primarily facilitated tie maintenance. These different types of social interaction then affected psychosocial outcomes through separate pathways: tie formation was directly associated with subjective health and well-being, while tie maintenance primarily supported relationship satisfaction, which subsequently contributed to improved health and well-being.

These findings have important implications for urban planning and policy, suggesting that different types of built environment interventions may be needed to support different forms of social interaction for enhancing public health and well-being.

### Limitations and future research

The study has some limitations that could be explored in future studies. First, the use of cross-sectional data limits the ability to establish causal relationships between the built environment, social interaction, and psychosocial outcomes. Second, the activity space models relied on self-reported locations and may have excluded routine but non-social destinations (e.g., grocery stores, pharmacies), potentially underestimating overall environmental exposure through daily mobility. Third, our classification of social interaction into tie formation and tie maintenance was based on reported social activity types, and did not directly measure tie strength—such as emotional closeness or duration of relationship—limiting the interpretation of social interaction outcomes, particularly when distinguishing weak from strong ties. Fourth, the analysis did not account for neighborhood self-selection, where individuals may choose residential environments based on their existing social network [71], potentially biasing observed associations. Fifth, qualitative features of the built environment, such as park design [38], the atmosphere and cleanliness of third places [72], and biodiversity or noise exposure in green spaces [73], were not included even though they may influence people’s behavior. Similarly, the walkability index did not capture qualitative aspects of the pedestrian environment, such as sidewalk conditions, street furniture, or feelings of safety, which could also affect social interaction [74, 75]. Sixth, this study did not account for other important factors that may influence social engagement, such as the relationship between social activity and travel behavior [76–78], as well as broader sociocultural dimensions like race and ethnicity [79], due to data limitations. Finally, the sample was limited to selected areas in Turku, Finland. Besides, the relatively low response rate (8.7%) of the PPGIS survey may have resulted in non-response bias, limiting the generalizability of the findings to other cultural or urban contexts.

Future research should address these limitations by adopting longitudinal or quasi-experimental designs to clarify causal relationships, and by incorporating both social and non-social destinations into activity space models to better capture environmental exposure. Measuring tie strength would help distinguish between different types of social interaction by accounting for weak and strong social ties, and would allow for examining their distinct impacts. Additionally, accounting for neighborhood self-selection would help clarify how individuals interact with their social and physical environments. Moreover, including qualitative features of built environment and travel behavior data could shed light on the experiential and behavioral mechanisms linking the built environment to social interaction. Finally, comparative studies across diverse cultural, racial, and urban contexts, supported by representative sampling, would enhance the generalizability and policy relevance of these findings.

## Conclusion

This study examined how residential urban form and detailed built environment characteristics influence two distinct types of social interaction—tie formation and tie maintenance—and how these in turn relate to psychosocial outcomes, including relationship satisfaction, subjective health, and subjective well-being. Drawing on survey data from Turku, Finland, and employing multidimensional activity space models, we assessed not only the residential neighborhood environment but also daily destinations and broader individualized activity ranges.

The findings revealed that urban residence plays a key role in supporting tie maintenance, with residents in compact urban areas more likely to sustain existing relationships within close proximity to home. However, urban residence alone was not associated with the formation of new ties, suggesting that simply living in an urban environment may not be sufficient to foster new social connections.

In contrast, specific features of the built environment indicated more nuanced patterns. Walkability around the home was positively associated with both tie formation and maintenance, underscoring the social value of pedestrian-friendly neighborhoods. Green spaces and parks, particularly those located near daily destinations or along the spatial range between home and destinations, were positively associated with tie formation. This suggests that outdoor green environments encountered during everyday routines may facilitate non-mandatory activities, such as spontaneous detours or additional trips, which in turn create opportunities for new social engagement. These findings indicate that not only residential neighborhoods but also daily destinations and broader activity ranges serve as important environments for fostering social interaction.

Furthermore, the two types of social interaction showed distinct pathways to psychosocial outcomes. Tie formation was found to influence subjective health and well-being directly, potentially through mechanisms such as expanded social horizons and exposure to new experiences. In contrast, tie maintenance contributed to well-being indirectly via relationship satisfaction, highlighting the emotional support and security provided by strong, existing relationships.

Taken together, these results emphasize the importance of distinguishing between types of social interaction and recognizing their differing spatial patterns. They carry valuable implications for urban planning and policy, suggesting that different features of the built environment may play distinct roles in fostering new social connections and supporting the maintenance of existing ones. To promote better health and well-being, as well as prevent loneliness, it is important to design environments that encourage both kinds of interaction in everyday life.

## Supplementary Information

Supplementary Material 1.

## Data Availability

The datasets generated and/or analyzed during the current study are not publicly available due to privacy issues regarding the study participants mapped home and daily activity point locations. However, the data are available from the corresponding author on reasonable request, but only after anonymization of sensitive material.

## References

[1] Peplau LA, Perlman D. Loneliness: a sourcebook of current theory, research, and therapy. New York: Wiley; 1982.

[2] Holt-Lunstad J, Smith TB, Baker M, Harris T, Stephenson D. Loneliness and social isolation as risk factors for mortality: a meta-analytic review. Perspect Psychol Sci. 2015;10(2):227–37. 25910392 10.1177/1745691614568352

[3] Manera KE, Smith B, Owen KB, Phongsavan P, Lim MH. Psychometric assessment of scales for measuring loneliness and social isolation: an analysis of the household, income and labour dynamics in Australia (HILDA) survey. Health Qual Life Outcomes. 2022;20:40. 10.1186/s12955-022-01946-6 35248075 10.1186/s12955-022-01946-6 PMC8897757

[4] Shankar A, Rafnsson SB, Steptoe A. Longitudinal associations between social connections and subjective wellbeing in the English longitudinal study of ageing. Psychol Health. 2015;30(6):686–98. 25350585 10.1080/08870446.2014.979823

[5] Buecker S, Horstmann KT. Loneliness and social isolation during the COVID-19 pandemic: a systematic review enriched with empirical evidence from a large-scale diary study. Eur Psychol. 2021;26(4):272–84.

[6] Hawkley LC, Cacioppo JT. Loneliness matters: a theoretical and empirical review of consequences and mechanisms. Ann Behav Med. 2010;40(2):218–27. 20652462 10.1007/s12160-010-9210-8 PMC3874845

[7] Lam J, Broccatelli C, Baxter J. Diversity of strong and weak ties and loneliness in older adults. J Aging Stud. 2023;64:101097. 36868610 10.1016/j.jaging.2022.101097

[8] Zhang X, Dong S. The relationships between social support and loneliness: a meta-analysis and review. Acta Psychol (Amst). 2022;227:103616. 35576818 10.1016/j.actpsy.2022.103616

[9] Diener E, Oishi S, Tay L. Advances in subjective well-being research. Nat Hum Behav. 2018;2(4):253–60. 30936533 10.1038/s41562-018-0307-6

[10] Mouratidis K. Urban planning and quality of life: a review of pathways linking the built environment to subjective well-being. Cities. 2021; 115:103229. 10.1016/j.cities.2021.10322934658478

[11] Umberson D, Montez JK. Social relationships and health: a flashpoint for health policy. J Health Soc Behav. 2010;51(SupplSuppl):S54–66. 20943583 10.1177/0022146510383501 PMC3150158

[12] Diener E, Seligman ME. Very happy people. Psychol Sci. 2002;13(1):81–4. 11894851 10.1111/1467-9280.00415

[13] Marans RW. Understanding environmental quality through quality of life studies: the 2001 DAS and its use of subjective and objective indicators. Landsc Urban Plann. 2003;65(1–2):73–83.

[14] Mouratidis K. Built environment and social well-being: how does urban form affect social life and personal relationships? Cities. 2018;74:7–20.

[15] Mouratidis K. Commute satisfaction, neighborhood satisfaction, and housing satisfaction as predictors of subjective well-being and indicators of urban livability. Travel Behav Soc. 2020;21:265–78.

[16] Granovetter M. The strength of weak ties. Am J Sociol. 1973;78:1360–80.

[17] Wellman B, Wortley S. Different strokes from different folks: community ties and social support. Am J Sociol. 1990;96(3):558–88.

[18] Smith KP, Christakis NA. Social networks and health. Annu Rev Sociol. 2008;34(1):405–29.

[19] Rivera MT, Soderstrom SB, Uzzi B. Dynamics of dyads in social networks: assortative, relational, and proximity mechanisms. Annu Rev Sociol. 2010;36(1):91–115.

[20] Kawachi I, Berkman LF. Social ties and mental health. J Urban Health Bull N Y Acad Med. 2001;78(3):458–67. 10.1093/jurban/78.3.458 PMC3455910 11564849

[21] Thoits PA. Mechanisms linking social ties and support to physical and mental health. J Health Soc Behav. 2011;52(2):145–61. 21673143 10.1177/0022146510395592

[22] Bower M, Kent J, Patulny R, Green O, McGrath L, Teesson L, et al. The impact of the built environment on loneliness: a systematic review and narrative synthesis. Health Place. 2023;79:102962. 36623467 10.1016/j.healthplace.2022.102962

[23] Leyden KM. Social capital and the built environment: the importance of walkable neighborhoods. Am J Public Health. 2003;93(9):1546–51. 12948978 10.2105/ajph.93.9.1546 PMC1448008

[24] Wood L, Frank LD, Giles-Corti B. Sense of community and its relationship with walking and neighborhood design. Soc Sci Med. 2010;70(9):1381–90. 20189699 10.1016/j.socscimed.2010.01.021

[25] Rogers SH, Halstead JM, Gardner KH, Carlson CH. Examining walkability and social capital as indicators of quality of life at the municipal and neighborhood scales. Appl Res Qual Life. 2010;6(2):201–13.

[26] Mazumdar S, Learnihan V, Cochrane T, Davey R. The built environment and social capital: a systematic review. Environ Behav. 2017;50(2):119–58.

[27] Ma L, Kent J, Mulley C. Transport disadvantage, social exclusion, and subjective well-being: The role of the neighborhood environment—evidence from Sydney, Australia. Journal of Transport and Land Use. 2018;11(1).

[28] van den Berg P, Sharmeen F, Weijs-Perrée M. On the subjective quality of social interactions: influence of neighborhood walkability, social cohesion and mobility choices. Transp Res Part A Policy Pract. 2017;106:309–19.

[29] Jacobs J. The death and life of great American cities. London: Jonathan Cape; 1961.

[30] Oldenburg R. The great good place: café, coffee shops, community centers, beauty parlors, general stores, bars, hangouts, and how they get you through the day. New York: Paragon House; 1989.

[31] Gardner PJ. Natural neighborhood networks — important social networks in the lives of older adults aging in place. J Aging Stud. 2011;25(3):263–71.

[32] Thompson S. Exploring the nature of third places and local social ties in High-Density areas: the case of a large Mixed-Use complex. Urban Policy Res. 2018; 36(3):304−18.

[33] Jing J, Dahlberg L, Canter D, Plater-Zyberk E, Collins T. The Role of Third Place concerning Loneliness in the Context of Ageing in Place: Three Neighbourhoods in Stockholm. Health & Social Care in the Community. 2024;4172682:1–16.

[34] Finlay J, Esposito M, Kim MH, Gomez-Lopez I, Clarke P. Closure of “third places”? Exploring potential consequences for collective health and wellbeing. Health Place. 2019;60:102225. 31622919 10.1016/j.healthplace.2019.102225 PMC6934089

[35] Kweon B-S, Sullivan WC, Wiley AR. Green common spaces and the social integration of inner-city older adults. Environ Behav. 1998;30(6):832–58.

[36] Hartig T, Mitchell R, de Vries S, Frumkin H. Nature and health. Annu Rev Public Health. 2014;35:207–28. 24387090 10.1146/annurev-publhealth-032013-182443

[37] Jennings V, Bamkole O. The relationship between social cohesion and urban green space: an avenue for health promotion. Int J Environ Res Public Health. 2019; 16(3): 452. 10.3390/ijerph16030452 30720732 10.3390/ijerph16030452 PMC6388234

[38] Peters K, Elands B, Buijs A. Social interactions in urban parks: stimulating social cohesion? Urban For Urban Green. 2010;9(2):93–100.

[39] Campbell LK, Svendsen ES, Sonti NF, Johnson ML. A social assessment of urban parkland: analyzing park use and meaning to inform management and resilience planning. Environ Sci Policy. 2016;62:34–44.

[40] Lund H. Testing the claims of new urbanism: local access, pedestrian travel, and neighboring behaviors. J Am Plann Assoc. 2003;69(4):414–29.

[41] Talen E, Koschinsky J. Compact, Walkable, Diverse Neighborhoods:Assessing Effects on Residents. Housing Policy Debate. 2014;24(4):717–50.

[42] Boessen A, Hipp JR, Butts CT, Nagle NN, Smith EJ. The built environment, spatial scale, and social networks: do land uses matter for personal network structure? Environ Plann B Urban Analytics City Sci. 2018;45(3):400–16.

[43] Jones M, Pebley AR. Redefining neighborhoods using common destinations: social characteristics of activity spaces and home census tracts compared. Demography. 2014;51(3):727–52. 24719273 10.1007/s13524-014-0283-z PMC4048777

[44] Kwan M-P. The uncertain geographic context problem. Ann Assoc Am Geogr. 2012;102(5):958–68.

[45] James P, Berrigan D, Hart JE, Hipp JA, Hoehner CM, Kerr J, et al. Effects of buffer size and shape on associations between the built environment and energy balance. Health Place. 2014;27:162–70. 24607875 10.1016/j.healthplace.2014.02.003 PMC4028172

[46] Zenk SN, Schulz AJ, Matthews SA, Odoms-Young A, Wilbur J, Wegrzyn L, et al. Activity space environment and dietary and physical activity behaviors: a pilot study. Health Place. 2011;17(5):1150–61. 21696995 10.1016/j.healthplace.2011.05.001 PMC3224849

[47] Browning CR, Soller B. Moving beyond neighborhood: activity spaces and ecological networks as contexts for youth development. Cityscape. 2014;16(1):165–96. 25105172 PMC4121985

[48] Wang Y, Kang C, Bettencourt LMA, Liu Y, Andris C. Linked activity spaces: embedding social networks in urban space. In: Helbich M, Jokar Arsanjani J, Leitner M, editors. Computational approaches for urban environments. Cham: Springer International Publishing; 2015. pp. 313–36.

[49] Hasanzadeh K, Broberg A, Kyttä M. Where is my neighborhood? A dynamic individual-based definition of home ranges and implementation of multiple evaluation criteria. Appl Geogr. 2017;84:1–10.

[50] Sharp G, Denney JT, Kimbro RT. Multiple contexts of exposure: activity spaces, residential neighborhoods, and self-rated health. Soc Sci Med. 2015;146:204–13. 26519605 10.1016/j.socscimed.2015.10.040

[51] Kestens Y, Thierry B, Shareck M, Steinmetz-Wood M, Chaix B. Integrating activity spaces in health research: comparing the VERITAS activity space questionnaire with 7-day GPS tracking and prompted recall. Spat Spatiotemporal Epidemiol. 2018;25:1–9. 29751887 10.1016/j.sste.2017.12.003

[52] Laatikainen TE, Hasanzadeh K, Kyttä M. Capturing exposure in environmental health research: challenges and opportunities of different activity space models. Int J Health Geogr. 2018;17(1):29. 30055616 10.1186/s12942-018-0149-5 PMC6064075

[53] Cagney KA, York Cornwell E, Goldman AW, Cai L. Urban mobility and activity space. Ann Rev Sociol. 2020;46(1):623–48.

[54] Statistics, Finland. Population structure 2025. https://pxdata.stat.fi/PXWeb/pxweb/en/StatFin/StatFin__vaerak/statfin_vaerak_pxt_11ra.px}

[55] Brown G, Kyttä M. Key issues and research priorities for public participation GIS (PPGIS): a synthesis based on empirical research. Appl Geogr. 2014;46:122–36.

[56] Eurostat_Quality of life indicators - social. interaction 2024. https://ec.europa.eu/eurostat/statistics-explained/index.php?title=Quality_of_life_indicators_-_social_interactions#Satisfaction_with_personal_relationships}

[57] WHO. Summary reports on proceedings minutes and final acts of the international health conference held in New York from 19 June to 22 July 1946. 1948.

[58] Zhang J, Yu Z, Zhao B, Sun R, Vejre H. Links between green space and public health: a bibliometric review of global research trends and future prospects from 1901 to 2019. Environ Res Lett. 2020; 15(6): 063001 . 10.1088/1748-9326/ab7f6436284641

[59] OECD. OECD Guidelines on Measuring Subjective Well-being. Paris: OECD Publishing; 2013. 24600748

[60] Hair JF, Hult GTM, Ringle CM, Sarstedt M, Danks NP, Ray S, editors. Evaluation of reflective measurement models. In: Hair JF, Hult GTM, Ringle CM, Sarstedt M, Danks NP, Ray S, editors. Partial least squares structural equation modeling (PLS-SEM) using R: a workbook. Cham: Springer International Publishing; 2021. p. 75–90.

[61] Byrne BM. Structural equation modeling with Amos: basic concepts, applications, and programming. New York, N.Y. ; Abingdon: Routledge; 2016. xx, 437 p. : ill. p.

[62] Hooper D, Coughlan J, Mullen MR. Structural equation modelling: guidelines for determining model fit. The Electronic Journal of Business Research Methods; 2008.

[63] Frank LD, Sallis JF, Saelens BE, Leary L, Cain K, Conway TL, et al. The development of a walkability index: application to the neighborhood quality of life study. Br J Sports Med. 2010;44(13):924–33. 19406732 10.1136/bjsm.2009.058701

[64] Taylor L, Hochuli DF. Defining greenspace: multiple uses across multiple disciplines. Landsc Urban Plann. 2017;158:25–38.

[65] Kyttä M, Broberg A, Haybatollahi M, Schmidt-Thomé K. Urban happiness: context-sensitive study of the social sustainability of urban settings. Environ Plan. 2015;43(1):34–57.

[66] Hasanzadeh K. IASM: individualized activity space modeler. SoftwareX. 2018;7:138–42.

[67] Zhou Q, Wang S, Liu Y. Exploring the accuracy and completeness patterns of global land-cover/land-use data in OpenStreetMap. Appl Geogr. 2022; 145: 102742. 10.1016/j.apgeog.2022.10274235469327

[68] Ngamaba KH, Panagioti M, Armitage CJ. How strongly related are health status and subjective well-being? Systematic review and meta-analysis. Eur J Public Health. 2017;27(5):879–85. 28957478 10.1093/eurpub/ckx081

[69] Reed S, Bohr J. The influence of local built environments on social wellbeing: a community’s experience with social Isolation, social Loneliness, and social belonging. Int J Community Well Being. 2020;4(3):393–413.

[70] Howell NA, Farber S, Widener MJ, Booth GL. Residential or activity space walkability: what drives transportation physical activity? J Transp Health. 2017;7:160–71.

[71] Guidon S, Wicki M, Bernauer T, Axhausen K. The social aspect of residential location choice: on the trade-off between proximity to social contacts and commuting. J Transp Geogr. 2019;74:333–40.

[72] Waxman L. The coffee shop: social and physical factors influencing place attachment. J Interior Des. 2006;31(3):35–53.

[73] Kajosaari A, Hasanzadeh K, Fagerholm N, Nummi P, Kuusisto-Hjort P, Kyttä M. Predicting context-sensitive urban green space quality to support urban green infrastructure planning. Landsc Urban Plan. 2024; 242: 104952. 10.1016/j.landurbplan.2023.104952

[74] Kuzuoglu S, Glover TD, Moyer L, Todd J. How built environment characteristics influence social interactions during neighbourhood walks among urban inhabitants. Int J Environ Res Public Health. 2024; 21(11): 1519. 10.3390/ijerph21111519 39595786 10.3390/ijerph21111519 PMC11593799

[75] Sonta A, Jiang X. Rethinking walkability: exploring the relationship between urban form and neighborhood social cohesion. Sustain Cities Soc. 2023; 99: 104903. 10.1016/j.scs.2023.104903

[76] Carrasco JA, Miller EJ. Exploring the propensity to perform social activities: a social network approach. Transportation. 2006;33(5):463–80.

[77] van den Berg P, Arentze T, Timmermans H. Location-type choice for face-to-face social activities and its effect on travel behavior. Environment and Planning B: Planning and Design. 2010;37(6):1057–75.

[78] Kim J, Rasouli S, Timmermans HJP. Social networks, social influence and activity-travel behaviour: a review of models and empirical evidence. Transp Rev. 2017;38(4):499–523.

[79] Anderson E. Black in white space: the enduring impact of color in everyday life. Chicago: University of Chicago Press; 2022.

