# Supplementary material for: The role of residential urban form and built environment in supporting social interaction, health, and well-being: a focus on forming and maintaining ties
Source: Int J Health Geogr. 2026 Jan 27;25:14. doi: 10.1186/s12942-026-00451-z (PMC12918495; doi:10.1186/s12942-026-00451-z)
Supplement: Supplementary file 1 — Supplementary Material 1. [file 12942_2026_451_MOESM1_ESM.docx]

**Appendix A. Validation of OSM-based Built Environment Indicators**

To assess the validity of OpenStreetMap (OSM) data used in this study, we compared OSM-derived land use classifications with two complementary reference datasets, summarized in Table A.1.

Table A.1. Description of reference datasets used for validation of OSM data

| Dataset | Year | Data type | Description |
| --- | --- | --- | --- |
| CORINE Land Cover dataset | 2018 | Vector (polygon) | Land-use classification data (vector polygons) with 44 categories across Europe. In this validation, classes 111–112 represent residential areas and 311–324 represent green areas. |
| SYKE NDVI (Normalized Difference Vegetation Index) dataset | 2021 | Raster  (10 m pixel) | NDVI maximum composite raster data (May–October 2021), measures vegetation density based on satellite imagery. Indicates vegetation density and health, with values scaled from −1 to 1. |

For the CORINE dataset, we evaluated the spatial agreement between OSM and CORINE classifications for residential and green areas. For the NDVI dataset, we identified green areas based on a threshold of NDVI > 0.6, which typically corresponds to dense and healthy vegetation.

All comparisons were conducted within the administrative boundary of the Turku region, as shown in Figure A.1. Following Zhou et al. (2022) [56], two metrics were used to evaluate the quality of OSM data:

Accuracy, defined as the proportion of OSM-labeled area that overlaps with the corresponding land use in the reference dataset.


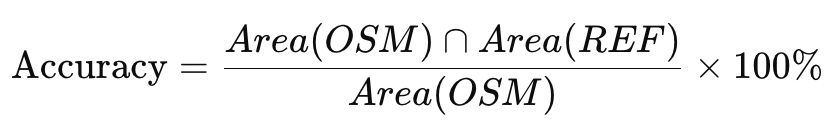


Completeness, defined as the proportion of the reference dataset that is covered by the OSM-labeled area.


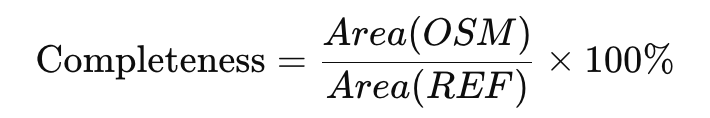


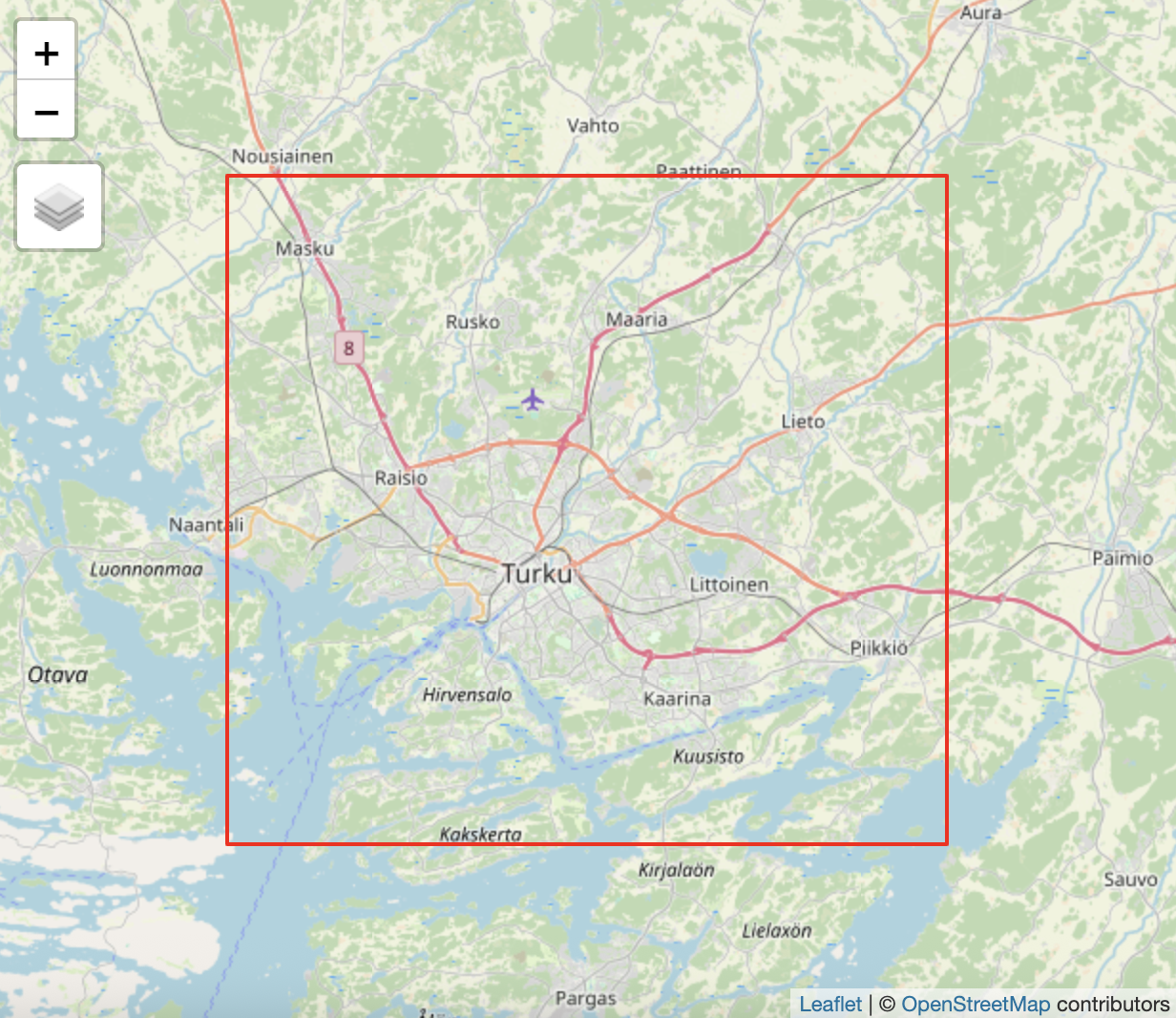


Figure A1. Spatial extent of the validation area in Turku

The results of the comparison are presented in Table A.2. Overall, OSM-derived land use classifications showed reasonably good agreement with both CORINE and NDVI datasets within the study area in Turku. Completeness values exceeding 100% (e.g., green areas compared with CORINE) indicate that the OSM-labeled areas were larger than those in the reference dataset. Figures A.2–A.4 provide visual illustrations of the spatial correspondence between OSM and the reference datasets.

Table A.2. Spatial agreement between OSM and reference datasets

| OSM land-use type | Reference data | Accuracy | Completeness |
| --- | --- | --- | --- |
| Residential area | CORINE (residential area) | 70.84% | 72.94% |
| Green area | CORINE (green area) | 68.25% | 114.54% |
| Green area | Syke NDVI (NDVI > 0.6) | 96.01% | 46.22% |

Figure A.2. Spatial comparison of residential land use between OSM and CORINE

Figure A.3. Spatial comparison of green area between OSM and CORINE

Figure A.4. Spatial comparison of green area between OSM and NDVI (NDVI > 0.6)

**Appendix B. Robustness Checks: Alternative Buffer Thresholds**

To assess the robustness of our SEM results, we tested different buffer distances (100 m, 200 m, and 300 m) around daily visited destinations in addition to the main threshold of 140 m. Table B.1 shows the results for Model (2.2) Home + daily visit point buffer, and Table B.2 shows the results for Model (2.3) Home range model. Both tables present standardized path coefficients from SEM analyses under each buffer setting.

Table B.1. Standardized path coefficients in SEM under different buffer thresholds

(Model 2.2: Home + daily visit point buffer)

|  | | *Buffer thresholds around daily visited destinations* | | | |
| --- | --- | --- | --- | --- | --- |
|  |  | *140m* | *100m* | *200m* | *300m* |
|  |  |  |  |  |  |
| *Path to* ***‘Tie formation’*** | |  |  |  |  |
|  | |  |  |  |  |
| *Built environment feature* | |  |  |  |  |
|  | Walkability | 0.172 | 0.150 | 0.149 | 0.150 |
|  | Third places density | 0.006 | 0.014 | -0.007 | -0.051 |
|  | Park ratio | 0.127* | 0.063 | 0.131* | 0.151** |
|  | Green space ratio | 0.180 ^a^ | 0.123 | 0.163 ^a^ | 0.163 ^a^ |
|  |  |  |  |  |  |
| *Path to* ***‘Tie maintenance’*** | |  |  |  |  |
|  | |  |  |  |  |
| *Built environment feature* | |  |  |  |  |
|  | Walkability | 0.115 | 0.173 | 0.093 | -0.012 |
|  | Third places density | -0.084 | -0.102 | -0.068 | -0.036 |
|  | Park ratio | 0.088 | 0.082 | 0.056 | 0.010 |
|  | Green space ratio | -0.077 | -0.033 | -0.097 | -0.196 |

*^a^: p < 0.10, *: p < 0.05, **: p < 0.01, ***: p < 0.001*

Table B.2. Standardized path coefficients in SEM under different buffer thresholds

(Model 2.3: Home range model)

|  | | *Buffer thresholds around daily visited destinations* | | | |
| --- | --- | --- | --- | --- | --- |
|  |  | *140m* | *100m* | *200m* | *300m* |
|  |  |  |  |  |  |
| *Path to* ***‘Tie formation’*** | |  |  |  |  |
|  | |  |  |  |  |
| *Built environment feature* | |  |  |  |  |
|  | Walkability | 0.092 | 0.097 | 0.126 | 0.140 |
|  | Third places density | -0.025 | -0.023 | -0.050 | -0.074 |
|  | Park ratio | 0.111* | 0.110* | 0.120* | 0.118* |
|  | Green space ratio | 0.140 | 0.139 | 0.166 ^a^ | 0.173 ^a^ |
|  |  |  |  |  |  |
| *Path to* ***‘Tie maintenance’*** | |  |  |  |  |
|  | |  |  |  |  |
| *Built environment feature* | |  |  |  |  |
|  | Walkability | 0.202 ^a^ | 0.121 | 0.117 | 0.086 |
|  | Third places density | -0.123 | -0.084 | -0.083 | -0.079 |
|  | Park ratio | 0.013 | 0.000 | 0.007 | -0.023 |
|  | Green space ratio | -0.022 | -0.083 | -0.079 | -0.108 |

*^a^: p < 0.10, *: p < 0.05, **: p < 0.01, ***: p < 0.001*
